# Supplementary material for: Factors related to time of stroke onset versus time of hospital arrival: A SITS registry-based study in an Egyptian stroke center
Source: PLoS One. 2020 Sep 11;15(9):e0238305. doi: 10.1371/journal.pone.0238305 (PMC7485782; doi:10.1371/journal.pone.0238305)
Supplement: Attachment — Submitted filename: Additional comments.docx [file pone.0238305.s001.docx]

Page 3:

- Did you obtain an official IRB approval?

Page 9:

- Please clarify the "off-hours" period.

Page 10:

- How did you select the periods of the day? Was this based on an analysis of number of ED consultations per hour, for example?
- Is the stroke frequency stablished as a relative measure in comparison with the total number of ED consultations?
- Please avoid non-relevant information for the methodology such us the use of a personal computer.
- Why was the Kolmorov-Smirnov test selected instead of the Shapiro-Wilks test?
- Was the skewness calculated?
- Why did you use the range and not the interquartile range as one of the dispersion measures?
- Do you consistently present frequencies in terms of both absolute and relative numbers?
- There are better options that Mann-Whintey's U test for comparing an ordinal dependent variable with a nominal independent variable.
- Did you use post-hoc tests?
- It seems to be a confusion between comparison, correlation, and association.
- How did you choose the cut-off for implementing the Fisher's exact test?

Page 11:

- How do you explain the small percentage of hemorrhagic stroke?
- Age is being presented with two different dispersion measures. Was it normal at the end?
- How do you explain the high frequency of diabetes? Do you have any comment about its attributable fraction for the Egyptian population?
- What do you think about this onset-to-door time in comparison with other populations?
- What was the distribution of patients among the two centers?
- Are there variables that significantly differ between those centers? Please describe the location and main characteristics of each center and access to resources and specialist physicians (vascular neurology, diagnostic neuroradiology, and interventional neuroradiology) in terms of quarters of the day and weekends. Who performs intravenous thrombolysis and mechanical thrombectomy?
- What do you think of the percentage of patients receiving thrombolytic therapy?
- What is the percentage of patients receiving mechanical thrombectomy?
- Please describe door-to-needle and door-to-groin periods, and factors associated as well with these in terms of day quarters.
- "Higher frequencies" at early morning and night can be due to a lower ED consultation rates at night and early morning. Are there significant differences in terms of relative frequencies (out of total ED patients per quarter of the day)? Or among centers?
- How are the results included in the study different among centers?
- How do you interpret all these findings beyond the statistical results?
- Please include data regarding medians and interquartile ranges and not only p values.
- About the stroke subtypes, findings might not be related to day quarters but to their specific epidemiology. I would recommend avoiding the calculation of spurious results if no biological or social plausibility is behind these assumptions.

Page 12:

- Discussion about circadian variations of stroke needs to be improved.
- There are several papers related to this issue, especially, in terms of biological hypothesis. Please discuss the pathophysiology beyond your findings.
- How is this paper useful for contributing to the discussion?
- How do you interpret these results in terms of what the literature has stablished?
- Additionally, it is important to clarify the availability of resources during the day and night, for properly discussing the findings.
- Specifically, which pre-hospital factors are more associated with receiving thrombolysis?
- Did you perform multivariate adjustment? Why not?
- Please re-analyze the data comparing working hours with off-work periods and clearly define this according to neurologists and interventional neuroradiologists shifts.
- How do you interpret the percentage of favorable outcomes in terms of other populations, the initial NIHSS, and previous mRS?
- What are the strengths, limitations, and future scope of this work?

Page 20:

- How were these variables chosen for being included in the figures? In its current shape, these figures involve several factors of the stroke phenomenon and might seems a little bit disorganized.
