# Supplementary material for: Factors related to time of stroke onset versus time of hospital arrival: A SITS registry-based study in an Egyptian stroke center
Source: PLoS One. 2020 Sep 11;15(9):e0238305. doi: 10.1371/journal.pone.0238305 (PMC7485782; doi:10.1371/journal.pone.0238305)
Supplement: Attachment — Submitted filename: RESPONSE TO REVIEWERS.docx [file pone.0238305.s002.docx]

Reviewer #1: MOST OF MY QUESTIONS ARE ANSWERED EXCEPT THE LAST 2

how much of the data is missing (particularly mRS at 30 days ) and what have they done for missing data

since the authors commented on good outcome at 30 days we need to see if their data is sound ,and what influenced the good outcome at 90 days in their cohort please discus in the discussion

We actually do not record mRS at 30 days as we are confined to data from SITS that includes mRS at 3 months only. The missing data at 3 months is only 344 patients.

could this be that inspite longer onset to door timings in the 3rd period , outcome was preserved due to prevalence of mild stroke ?

this assumption is actually sound and we added a paragraph with this meaning in discussion:

“And despite that onset to door time was more prolonged with onset of stroke in the 3^rd^ quarter (evening), yet the 3 months outcome did not differ from other quarters, possibly due to less stroke severity in the 3^rd^ quarter as shown by a significantly lower NIHSS.”

i also suggest adding missing data to the limitation of the study

done

Reviewer #2: 733/5000

Introducing the context of treatment availability and center logistics helps the reader better understand the landscape of stroke management at their centers.

We added this paragraph to introduction: “The study has been conducted in the two certified stroke centers of Ain Shams University hospitals, both of which apply the acute stroke management protocol approved by the stroke chapter of the Egyptian Society of Neurology, Psychiatry, and Neurosurgery to be the standard protocol for Egyptian stroke units. And both have CT scan and MRI devices available over 24 hours. Thrombolytic therapy is available and covered either by medical insurance or by the reimbursement from the Ministry of Health.”

Among the pre-hospital factors, it would be interesting to note, in addition to the level of knowledge of the symptoms by the population, other items, such as the method of arrival at the hospital (ambulance, private car or public transport), if they have pre-notification by the ambulances.

Regarding method of arrival at hospital, we are studying these factors in another article, about causes of pre-hospital delay, that is still under submission.

It would also be important to know the percentage of patients with wake-up stroke in each quarters.

Also the percentage of the wake up stroke is added to the limitation of this study.

Additionally, in the results section in the paragraph on factors related to stroke onset, the wording could be improved for better understanding of the reader.

Done
